# The Co-Association of Enterobacteriaceae and *Pseudomonas* with Specific Resistant Cucumber against *Fusarium* Wilt Disease

**DOI:** 10.3390/biology12020143

**Published:** 2023-01-17

**Authors:** Yu-Lu Zhang, Xiao-Jing Guo, Xin Huang, Rong-Jun Guo, Xiao-Hong Lu, Shi-Dong Li, Hao Zhang

**Affiliations:** 1 Institute of Plant Protection, Chinese Academy of Agricultural Sciences, Beijing 100193, China; 2 College of Plant Protection, Jilin Agricultural University, Changchun 130118, China

**Keywords:** cucumber *Fusarium* wilt (CFW), *Fusarium oxysporum* f. sp. *cucumerinum* (*Foc*), resistant and susceptible cultivars, root microbiota, suppressive functions

## Abstract

**Simple Summary:**

Pathogenic attack is a serious biotic stress that negatively affects agricultural and food production in the world. Currently, the use of beneficial plant bacteria for healthy plant growth is attractive due to the demand for eco-friendly and sustainable agriculture. In this study, the culture-free analyses of the root bacterial communities of six cucumber cultivars were compared to define the important differential bacteria associated with the resistant and moderately resistant cultivars against *Fusarium oxysporum* f. sp. *cucumerinum*, and the disease-suppressive function of the differential bacterium alone or in a complex were tested in a pot experiment. Our results highlighted that Enterobacteriacea/*Pantoea*, Enterobacteriaceae/*Cronobacter*, and Pseudomonadaceae/*Pseudomonas* were important differential phyla/genera associated with specific resistant cultivars, while *Massilia* only differed in response to pathogenic attack. The pot experiments confirmed that the differential bacteria complexes *Pantoea* + *Pseudomonas* and *Cronobacter* effectively alleviated disease occurrence. In conclusion, we provide supporting evidence on the potential of root bacteria from resistant cultivars to be regulated or applied to control cucumber wilt disease and promote healthy cucumber growth under pathogenic stress.

**Abstract:**

The root microbiota contributes to the plant’s defense against stresses and pathogens. However, the co-association pattern of functional bacteria that improves plant resistance has not been interpreted clearly. Using Illumina high-throughput sequencing technology, the root bacterial community profiles of six cucumber cultivars with different resistance in response to the causative agent of cucumber *Fusarium* wilt (CFW), *Fusarium oxysporum* f. sp. *cucumerinum* (*Foc*), were analyzed. The principal coordinate analysis indicated that the interactions of the cultivars and pathogens drove the cucumber root bacterial communities (*p* = 0.001). The resistance-specific differential genera across the cultivars were identified, including *Massilia* in the resistant cultivars, unclassified Enterobacteriaceae in resistant CL11 and JY409, *Pseudomonas* in JY409, *Cronobacter* in moderately resistant ZN106, and unclassified Rhizobiaceae and *Streptomyces* in susceptible ZN6. The predominant root bacterium *Massilia* accounted for the relative abundance of up to 28.08–61.55%, but dramatically declined to 9.36% in *Foc*-inoculated susceptible ZN6. *Pseudomonas* ASV103 and ASV48 of Pseudomonadaceae and *Cronobacter* ASV162 of Enterobacteriaceae were consistently differential across the cultivars at the phylum, genus, and ASV levels. Using the culture-based method, antagonistic strains of Enterobacteriaceae with a high proportion of 51% were isolated. Furthermore, the bacterial complexes of *Pantoea dispersa* E318 + *Pseudomonas koreensis* Ps213 and *Cronobacter* spp. C1 + C7 reduced the disease index of CFW by 77.2% and 60.0% in the pot experiment, respectively. This study reveals the co-association of specific root bacteria with host plants and reveals insight into the suppressing mechanism of resistant cultivars against CFW disease by regulating the root microbiota.

## 1. Introduction

Plants are colonized by the massive diversity of microorganisms, which fulfills important functions involving plant growth, nutrient acquisition, as well as defense against biotic and abiotic stresses through a combination of the host and host-associated microbiomes [1,2]. During the whole growing stage, a fraction of microbes maintain high abundances throughout plant development and functions through direct and indirect beneficial effects on hosts [3,4] regardless of the variation of plant microbiomes with the soil type and plant growing stage [5,6]. This kind of co-association of a plant microbiome with a domesticated cultivar or breeding history has been evidenced on many plants, such as lettuce, common bean, sunflower, sugarbeet, rice, barley, wheat, tomato, apple, etc. [7,8,9,10,11,12,13,14,15]. However, in addition to the plant domestication of specific microbes, it is important to understand the association of root microbiota with cultivar traits, such as disease resistance, cold or high-temperature tolerance, and flooding or drought resistance which are selected or acquired during the breeding process [16,17].

The selection of bacterial taxa by resistant or susceptible cultivars was studied in previous research. Weinert et al. [18] revealed that Pseudomonadales, Actinomycetales, and Enterobacteriales are differential taxa among three potato cultivars, although they account for small proportions of the bacterial community. Mendes et al. [13] reported that the *Fusarium oxysporum* (*Fo*)-resistant cultivar of common bean recruits Pseudomonadaceae, Bacillaceae, Solibacteraceae, and Cytophagaceae in the rhizosphere, and their abundances are positively correlated to *Fo* resistance. Kwak et al. [19] found that the tomato wilt-resistant variety Hawaii 7996 recruited Flavobacterium in the roots and suppressed the wilt disease. Moreover, Liu et al. [20] found that *Pseudomonas*, *Arthrobacter*, and *Bacillus* are enriched in the rhizosphere of resistant sugarbeet and inferred that the rhizosphere bacteria contributed to cultivar resistance. In addition to the rhizosphere microbiome, plant seeds or endophytic microbiota are also closely interacted and co-evolved with the host; for example, the evolutionary distance of *Malus* species and their microbiomes are significantly correlated [7].

The cucumber is a worldwide cultivated vegetable, which is frequently threatened by the cucumber *Fusarium* wilt (CFW) disease caused by *Fusarium oxysporum* f. sp. *cucumerinum* (*Foc*). Studies on the association of bacteria with cucumber cultivars have focused on rhizospheric bacteria, and the results are inconsistent. Nitrobacteria, actinomycetes, and arbuscular mycorrhiza fungi (AMF) are enriched in the rhizospheres of resistant cultivars [21,22], while Comamonadaceae and Xanthomonadaceae are enriched in the rhizospheres of susceptible cultivars [23]. In addition to a field survey, cultivar resistance to CFW was also evaluated in artificially infested soil under unified conditions to avoid the variation of pathogen populations and interference with other pathogens, and cultivar resistance was found to be mainly determined by the vertical genetic characteristics of the host and the root-associated microbiota [24]. However, how the root microbiota is associated with cultivar resistance has not been clearly interpreted yet.

In this study, six cucumber cultivars were collected, including two southern cultivars, CL11 and EZZ, with unknown resistance to CFW. CL11 is tolerant to low temperatures [25], resistant ZN106 is tolerant to high temperatures and can be cultivated both in northern and southern China [26]. The two northern cultivars, JY 409 and JY35, have high and moderate CFW resistance, respectively [27,28], and the northern cultivar ZN6 is susceptible to CFW [29]. To reveal the relationship between cultivar resistance and root microbiota, the resistance levels of these cultivars to CFW were first evaluated in sterilized soil with *Foc* inoculation. Then, their root bacterial communities were analyzed, and the important bacteria related to resistant cucumber against CFW were identified and functionally verified.

## 2. Materials and Methods

### 2.1. Cucumber Cultivars

Six cucumber cultivars were used in this study. ZN106 and ZN6 were provided by the Institute of Vegetables and Flowers from the Chinese Academy of Agricultural Sciences, JY409 and JY35 were provided by the Tianjin Kernel Vegetable Research Institute, and CL11 and EZZ were provided by the Horticulture Research Institute from the Sichuan Academy of Agricultural Sciences. After drying at 68 °C for 3 h, the seeds were surface-disinfected in 3% NaClO for 5 min, washed with sterile water 5 times, and germinated on sterile wet filter paper at 25 °C for 24–48 h.

### 2.2. Preparation of the Foc Spore Suspension

Strain M7 of *Foc* (ACCC 39679 from the Agriculture Culture Collection of China) was isolated from cucumber roots. Fresh mycelia were inoculated into liquid PDB (potato 200 g L^−1^, glucose 20 g L^−1^) and grown at 28 °C with constant agitation at 180 rpm for 96 h. The spores were then collected by filtering through a sterilized miracloth (Merck-Millipore, 475855-1R) and enumerated under an Olympus microscope (BX41, Tokyo, Japan) using a hemocytometer.

### 2.3. Resistance Evaluation and Root Sampling

The pot experiment was carried out in the greenhouse of the Institute of Plant Protection in the Chinese Academy of Agricultural Sciences (Beijing, China). Potting soil provided by Qingdao Lvsheng Biotechnology Co. Ltd. (organic content 37%, pH 6.8) was sieved and autoclaved. Each pot (7 cm-in-diameter × 7 cm-in-height) containing 160 g of soil was initially watered to 50% field capacity and sprayed with 2 g of pre-inoculated soil containing *Foc* spores at a final concentration of 3 × 10^5^ spores g^−1^. Six germinated seeds were sowed and covered with 2 g of autoclaved potting soil. Each cultivar had six replicate pots, and the non-*Foc* (mock) inoculated pots were taken as the control. All the cucumbers were grown at 26–28 °C for 2 weeks and irrigated with 15 mL of water every 2 days to keep the water content at 50–55%. The disease incidence, disease index (DI), and disease symptoms were investigated in the second week, as previously described [30]. All data were statistically analyzed using SPSS v20.0, and significant differences were determined using Duncan’s new multiple-range test or a *t*-test at *p* < 0.05. The resistant levels of the cultivars were determined according to the DI value, i.e., resistant, 0 < DI ≤ 20; moderately resistant, 20 < DI ≤ 40; and susceptible, 40 < DI ≤ 75.

The roots of the 2 week-old cucumber seedlings were rinsed with sterile water, dried on sterile filter paper, and cut into 1 cm segments with sterile scissors. Approximately 200 mg of root segments of each cultivar were combined, grounded into powder with 60 mg of sterilized quartz sand in liquid nitrogen, and suspended in 4 mL of phosphate buffer (pH 5.5). After filtration through sterile Whatman filter paper (1002-90, 8 µm; 1006-110, 3 µm) and centrifugation at 4 °C, 10,000 rpm for 10 min, the residues were used for the DNA extraction and isolation of the root bacteria. Each treatment was performed in triplicates.

### 2.4. DNA Extraction and Illumina Miseq Sequencing

Total DNA was extracted using the FastDNA^®^ SPIN kit for soil (MP Biomedicals, LLC, Irvine, CA, USA). The DNA quality was checked via electrophoresis in 1% agarose gel with a spectrophotometer (A_260/280_). The DNA sample was stored at −20 °C before the analysis. The V5–V7 hypervariable region of the bacterial 16S rRNA gene spanning ~394 bp was amplified with a two-step PCR amplification method using two primer sets 799F (5′-AACMGGATTAGATACCCKG-3′) and 1392R (5′-ACGGGCGGTGTGTRC-3′) [31,32] and 799F and 1193R (5′-ACGTCATCCCCACCTTCC-3′) [8,33], respectively. The first-step amplification conditions were initial denaturation at 95 ℃ for 3 min, followed by 27 cycles of denaturing at 95 ℃ for 30 s, annealing at 55 °C for 30 s, and extension at 72 ℃ for 45 s, and another extension at 72 ℃ for 10 min. The second-step amplification conditions were similar to those of the first step except for 15 amplification cycles. The 20 μL PCR mixtures contained 5 × *TransStart* FastPfu buffer 4 μL, 2.5 mM dNTPs 2 μL, forward/reverse primer (5 μM) 0.8 μL, *TransStart* FastPfu DNA Polymerase 0.4 μL, BSA 0.2 μL, and template DNA 10 ng. The PCR reactions were performed in triplicates. The PCR products were extracted from the 2% agarose gel and purified using the AxyPrep DNA Gel Extraction Kit (Axygen Biosciences, Union, CA, USA) according to the manufacturer’s instructions. After the quantification using QuantiFluor™-ST (Promega, Madison, WI, USA), the PCR products were subjected to high-throughput sequencing on the Illumina Miseq platform according to the standard protocol provided by Majorbio Bio-Pharm Technology Co. Ltd. (Shanghai, China). Sterile RNase-free water was used as the negative control of each PCR run. The raw reads were deposited into the NCBI Sequence Read Archive (SRA) database under the accession number SRP385205.

### 2.5. Bioinformatic Analyses of the Amplicon Sequence Dataset

The partial 16S rRNA gene sequences were processed using the Illumina Analysis Pipeline v4.0 developed by Majorbio. The demultiplexed raw reads were subjected to quality filtering with fastp v0.20.0 [34] based on the sequence length and quality. Briefly, the reads were truncated to a length of 300 bp, and only the reads with a quality score of >20 and no ambiguous bases were retained for further analysis. The reads that overlapped more than 10 bp were merged with FLASH v1.2.11 [35], and the read pairs that could not be assembled were discarded. After distinguishing the sequences of each sample according to the barcode and primers, the sequence direction was adjusted, the exact barcode was matched, and the tag sequences and chimeric sequences were discarded. Then the sequences were denoised using DADA2 [36] to obtain the amplicon sequence variants (ASVs) plugin in the Qiime2 v2020.2 [37] pipeline with the recommended parameters. To minimize the effects of sequencing depth on the alpha and beta diversity, the number of sequences from each sample was rarefied to 9921. The taxonomy of each representative sequence of ASV was annotated using the Naive Bayes consensus taxonomy classifier against the SILVA 16S rRNA database (v138) with a confidential threshold of 70%. The ASVs identified as mitochondria, chloroplasts, or cyanobacteria were removed.

The bacterial phyla, families, and genera with relative abundances over 1.0%, 0.5%, and 1.0% were identified, respectively. To determine the abundance shifts, the top 20 bacterial genera were illustrated with a heatmap produced using Python 2.7 in R v3.3.1. To determine the factors driving the community shift, a principal coordinate analysis (PCoA) [38] was performed to illustrate the clustering of the bacterial communities based on the Bray-Curtis distance of the ASVs using the Vegan package. A permutational multivariate analysis of variance (PERMANOVA) was used to analyze the contribution of the cultivar and *Foc* spiking to the root bacterial community [39]. Differential bacterial genera affected by the cucumber cultivar and *Foc* spiking were identified by the comparison of their average relative abundances with the Kruskal-Wallis H test and confirmed with the false discovery rate (FDR) (*p* ≤ 0.05). Furthermore, differential bacterial ASVs across the cultivars were identified and selected for further tests.

### 2.6. Isolation and Identification of Culturable Endophytic Bacteria

Two methods were used to isolate the root bacteria. Following the conventional strategy, aliquots of 20 µL cucumber root residues and their 10-fold dilutions were plated on 1/10 TSA medium (tryptone 15 g L^−1^, yeast extract 0.5 g L^−1^, KH_2_PO_4_ 0.5 g L^−1^, NaCl 25 g L^−1^, agar 18 g L^−1^, pH 7.0; [40,41]) supplemented with 100 μg mL^−1^ Tebuconazole, and incubated at 28 °C up to 7 days. Single colonies of different morphologies were picked up and purified on 1/10 TSA plates. In addition, the bacterial band method [30,42] was used, as shown in Figure S1. Aliquots (10 μL) of cucumber root residues and their 10-fold dilutions were grown on WA-N plates (1.8% agar) using a sterile cell spatula as a bacterial zone of 1.8 cm in width at the center of a 9 cm-in-diameter plate and incubated at 26 °C for 3 days, followed by the inoculation of a 5 mm-in-diameter *Foc* plug 2.5 cm away from the bacterial zone. The WA-N plates without bacterial streaking were taken as controls. After incubation at 20 °C for 15 days, bacteria forming clear zones inhibiting the *Foc* growth or attaching to *Foc* hyphae were picked and purified on 1/10 TSA plates. Each root sample had three replicates, and the purified isolates were stored in 30% sterile glycerol at −80 °C.

For the molecular identification, bacterial isolates grown in liquid TSA medium with constant agitation at 180 rpm at 28 °C overnight were subjected to DNA extraction using the TIANamp bacteria DNA kit (DP302; TIANGEN, Beijing, China) and PCR amplification with the universal primers 27F and 1492R [43]. The amplicons were confirmed in a 1.2% agarose gel and sequenced by Tsingke Biotech (Beijing, China) and Sangon Biotech (Shanghai, China). The top hits (>97% sequence identity) of an NCBI BLASTn search (http://blast.ncbi.nlm.nih.gov, accessed on 14 December 2022) were used to assign each isolate to the genus level. The 16S rRNA sequences of the bacterial isolates were also compared with those obtained from the Illumina sequence analysis and assigned to their representative ASVs.

### 2.7. Antagonism Test In Vitro

The antagonistic activity of each bacterial isolate was tested using the conventional pair-culturing method. Briefly, a fresh plug of *Foc* was placed on the center of a 9-cm-in-diameter petri dish containing 18 mL of half-strength (1/2) PDA (potato 100 g L^−1^, glucose 10 g L^−1^, and agar 18 g L^−1^), and four different bacterial isolates were streak-inoculated at four corners in the distance of 2 cm to the center. Non-bacterial inoculation served as the control. After incubation at 28 °C for 5 days, the antagonistic ability of each isolate was assessed by measuring the radii of the *Foc* colonies. The inhibition rate was calculated following the formula R1 − R2/R1, where R1 and R2 were the radii of the *Foc* colonies (mm) without and with the antagonistic bacteria, respectively [30].

### 2.8. Suppressive Ability of the Selected Bacteria on CFW in the Pot Experiment

The phylogenetic tree of differential bacteria *Cronobacter* C1 and C7 (ZN106, ASV162), *Pantoea* P304 (CL11, ASV343), *Pantoea* E318 (JY409, ASV343), *Pseudomonas* Ps213 (JY409, ASV466) were constructed using MEGA 11.0 software and identified based on the homology. The individual bacterium of P304, E318, and Ps213 and the bacterial complexes of strains E318 and Ps213 (BCX1, 1:1) and strains C1 and C7 (BCX2, 1:1) were used in the pot experiment. Pots (8.8 cm × 8.8 cm × 9.6 cm) containing 90 g of autoclaved soil with a water content of 50% were covered with 90 g of bacteria inoculated soil at the concentration of 10^6^ CFU g soil^−1^ with the same water content. As shown in Figure S2, two planting furrows were formed at a distance of 1.5 cm from the pot edge, and three germinated cucumber seeds with 1 cm hypocotyl were sowed and covered with a thinner layer of the same bacteria-inoculated soil. The soil without bacterial inoculation was taken as the control. Each treatment had five replicates. The pots were covered with plastic to retain the soil’s moisture and were irrigated once every two days with 20 mL of water to keep the soil moisture at 52–55% in the first week. One week later, six *Foc* plugs 5 mm in diameter were down-inoculated on the soil surface, 1 cm away from each seedling. The pots were put in a growth chamber at 26 ℃ (16 h day/8 h night, with a light intensity of 20 klx, and relative humidity of 95%). The pots were irrigated with 20 mL of water once a day during the second and third weeks. The cucumber seedlings were then subjected to the determination of disease severity as described by Sun et al. [30]. The control efficacy was calculated based on the disease indices of treatments against the control. The data were analyzed using SPSS 20.0, and significant differences were determined using Duncan’s new multiple-range test at *p* ≤ 0.05.

## 3. Results

### 3.1. Resistance Levels of the Cucumber Cultivars

The six cucumber cultivars demonstrated different resistance against CFW in the pot experiment. As shown in Table S1, CL11 and EZZ showed the highest resistance to *Foc* infection without any wilt symptoms on the seedlings. JY409 was also resistant according to the DI criterion (less than 10). JY35 and ZN106 were moderately resistant with DIs of 15.27 to 23.61, and ZN6 was susceptible with a DI of up to 51.39.

### 3.2. Analyses of the Root Bacterial Microbiota

Single reads of 10,161 to 24,388 were obtained from 36 root samples, and a total of 9921 tags were yielded after the evenness analysis. After the taxonomic assignment and removing the ASVs of singletons, mitochondria, and chloroplasts, 548 distinct ASVs belonging to 4 phyla, 25 families, and 26 genera were identified.

The ɑ-diversity of the root bacterial microbiota of six cucumber cultivars with and without *Foc* inoculation is shown in Table 1. The Goods’ coverage data of all samples were 1.00, indicating that the sequences were qualified for the diversity analysis. The ɑ-diversity demonstrated with Good’s coverage, and the Chao, Shannon and Simpson indices related to the bacterial richness and diversity had no significant difference among the samples (*p* > 0.05). This suggested that the root bacterial microbiota across the cucumber cultivars and treatments were similar in their ɑ-diversity.

**Figure 1:**
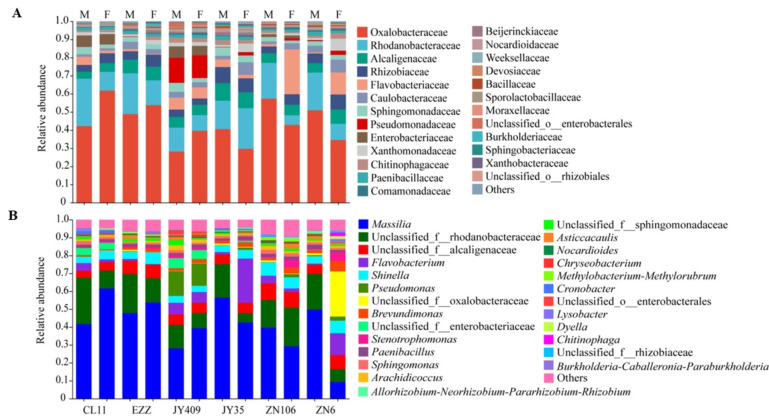
Taxonomic profiling of the bacterial communities in cucumber root across the cultivars and treatments at the (**A**) family and (**B**) genus levels. The families and genera with a proportion of less than 0.5% and 1.0%, respectively, were combined into the group “Others”. Cucumber cultivars CL11, EZZ, and JY409 are resistant, JY35 and ZN106 are moderately resistant, and ZN6 is susceptible. M and F indicate mock and *Foc* inoculation, respectively.

The root microbiota assembly of cucumber was dominated by four phyla across the cultivars, in which Proteobacteria, Bacteroidota, Firmicutes, and Actinobacteria accounted for 71.41–94.59%, 2.15–25.81%, 1.87–4.00%, and 0.21–1.57% of the bacterial communities, respectively. Upon *Foc* inoculation, the Proteobacterial abundances of ZN106 and ZN6 decreased, while Bacteroidota increased (Figure S3). At the family level (Figure 1A), 25 families with an abundance of over 0.5% were detected altogether. Oxalobacteraceae was the most predominant family across the cultivars, accounting for 28.21–61.80% of the root bacteria, followed by Rhodanobacteraceae (5.27–26.10%), Alcaligenaceae (3.83–9.73%), and Rhizobiaceae (3.52–8.78%). Burkholderiaceae and unclassified_o_Enterobacterales were rare bacterial families, only accounting for small proportions of 0–1.28% and 0–2.54%, respectively. Several bacterial families were cultivar-specific, such as Enterobacteriaceae, which predominated in CL11, JY409, and ZN106 (1.33–6.24% vs. 0–0.18% of other samples), and Pseudomonadaceae, which predominated in JY409 (12.51% and 13.86% vs. 0–2.32% of other samples). Upon *Foc* inoculation, the proportions of Oxalobacteraceae, Rhodanobacteraceae, and Flavobacteriaceae had obvious changes. The abundances of Oxalobacteraceae were increased in resistant CL11, EZZ, and JY409 but decreased in moderately resistant JY35 and ZN106 and susceptible ZN6. In contrast, the abundances of Rhodanobacteraceae were decreased in all cultivars except for JY35, and Flavobacteriaceae were enriched in ZN106 and ZN6 with proportions of 24.75% and 12.17% (vs. <1.31%). At the genus level (Figure 1B, Table S2), *Massilia* was predominant (28.08–61.55%) in all samples except for *Foc*-inoculated ZN6 (9.36%), and the other dominant genera were unclassified_f_Rhodanobacteraceae (5.17–26.02%), unclassified_f_Alcaligenaceae (3.83–9.33%), *Flavobacterium* (0.40–24.75%), and *Shinella* (3.83–9.33%). *Chitinophaga* and *Dyella* were rare genera with proportions of 0–1.63% and 0–2.01%, respectively. Cultivar-specific genera were also identified, including the unclassified_f_ Enterobacteriaceae in resistant CL11 and JY409, *Cronobacter* in CL11 and ZN106, *Pseudomonas* in JY409, *Methylobacterium*-*Methylorubrum*, *Nocardioides,* and *Bacillus* in JY35, and *Asticcacaulis* in EZZ, JY409, and ZN6. Upon *Foc* inoculation, the abundance changes of *Massila*, unclassified_f_Rhodanobacteraceae, and *Flavobacterium* showed similar patterns to their corresponding families Oxalobacteraceae, Rhodanobacteraceae, and Flavobacteriaceae, respectively, except that the proportion of *Massila* dramatically decreased in susceptible ZN6. Interestingly, the abundance of unclassified_f_Oxalobacteraceae was increased significantly in susceptible ZN6 from 1.12% to 25.22%. Both *Massilia* and unclassified_f_Oxalobacteraceae belong to Oxalobacteraceae, and their abundance sum in each cultivar showed a similar changing pattern with Oxalobacteraceae (Table S3), suggesting that the bacterial community shifts at the family and genus levels were consistent.

**Figure 2:**
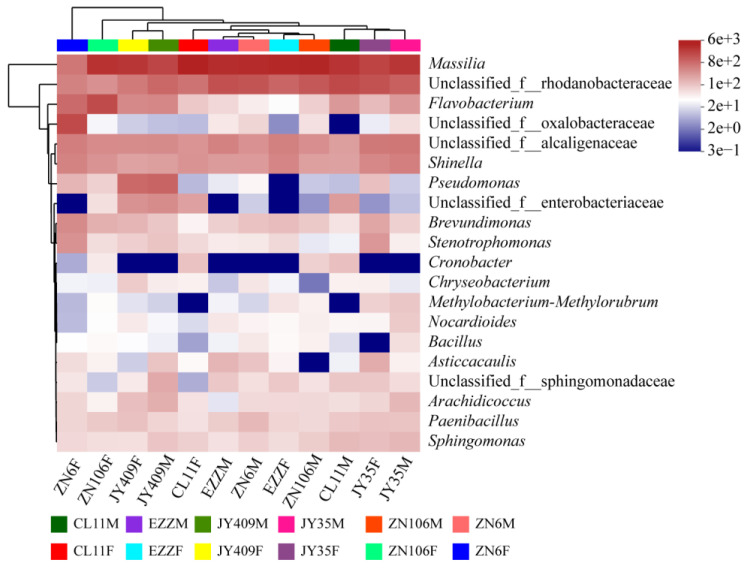
Heatmap analysis of the top 20 bacterial genera of cucumber cultivar root microbiota. M and F following the cultivar names indicate mock and *Foc* inoculation, respectively.

The hierarchical heatmap analysis of the top 20 dominant genera (Figure 2) illustrated that the root bacterial communities were related to the treatments (mock and *Foc* inoculation) and cucumber cultivars. The communities of inoculated ZN6 samples were separated from the other samples. *Massilia*, unclassified_f_Rhodanobacteraceae, Alcaligenaceae, and *Shinella* were predominant in all root samples, and *Flavobacterium*, *Brevundimonas*, *Stenotrophomonas*, *Paenibacillus*, *Sphingomonas*, *Arachidicoccus,* and unclassified_f_Sphingomonadaceae were also detected in all samples but with low abundances. Some root bacteria showed similar abundance profiles across cultivars, such as unclassified_f_Alcaligenaceae and *Shinella*, *Brevundimonas*, and *Stenotrophomonas*, while the most predominant *Massilia* and unclassified_f_Rhodanobacteraceae had cultivar-specific patterns far different from others. The results indicated that the root microbiota of different cucumber cultivars had specific assemblies, and those differential ones across cultivars or upon *Foc* spiking might be the important bacteria that contribute to cucumber resistance against *Foc*.

### 3.3. Factors Affecting the Root Bacterial Microbiota

**Figure 3:**
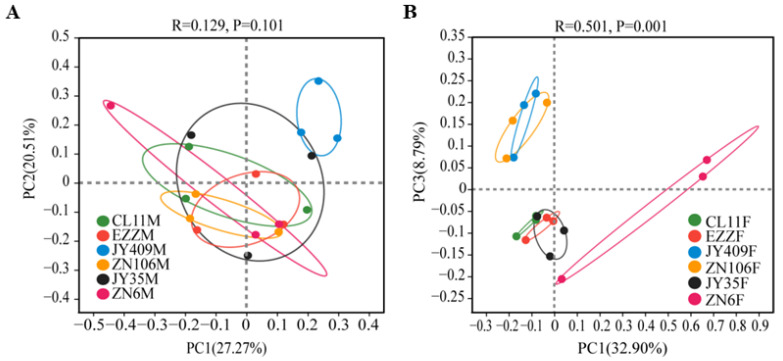
Comparison of the bacterial community structures of cucumber root across the cultivars and treatments using the PCoA analysis. (**A**) Mock and (**B**) *Foc* inoculation.

Variations in the bacterial microbiota across six cucumber cultivars and two treatments were analyzed using the PCoA ordination and PERMANOVA analyses. The PCoA ordination indicated that the root bacterial microbiota of different cultivars with mock inoculations clustered together (Figure 3A, *p* = 0.101), but separated into three clusters (JY409 and ZN106; CL11, EZZ, and JY35; ZN6) upon *Foc* inoculation (Figure 3B, *p* = 0.001). It meant that different cucumber cultivars had similar root bacterial communities but varied in their responses to pathogens. The shift of the root microbiota was closely correlated to some cultivar varieties, and the root microbiota of all resistant and moderately resistant cultivars were separated from susceptible ZN6. The co-association was further validated with the PERMANOVA analyses of 18 samples in each treatment group and 36 samples in combination. No significant difference (*p* = 0.156) was observed in the root microbiota among the cultivars with mock inoculation, but a very significant difference (*p* = 0.001) was detected in the *Foc*-inoculated group (Table S4). When combining the mock and *Foc* inoculation groups, significant differences (*p* = 0.001 and *p* = 0.04) were detected among the cultivars and between the treatment groups, respectively. Further comparison analyses using ANOSIM (Table S5) showed that *Foc* inoculation had no significant effect (*p* > 0.05) on the root bacterial microbiota of each cultivar. It suggested that the 2-week co-culture with *Foc* exerted little impact on the root bacterial microbiota of the cultivar. Instead, the resistant traits of the cultivars played key roles in shaping the composition of the root bacterial communities in response to *Foc* inoculation.

### 3.4. Identification of Differential Root Bacteria across Cultivars

**Figure 4:**
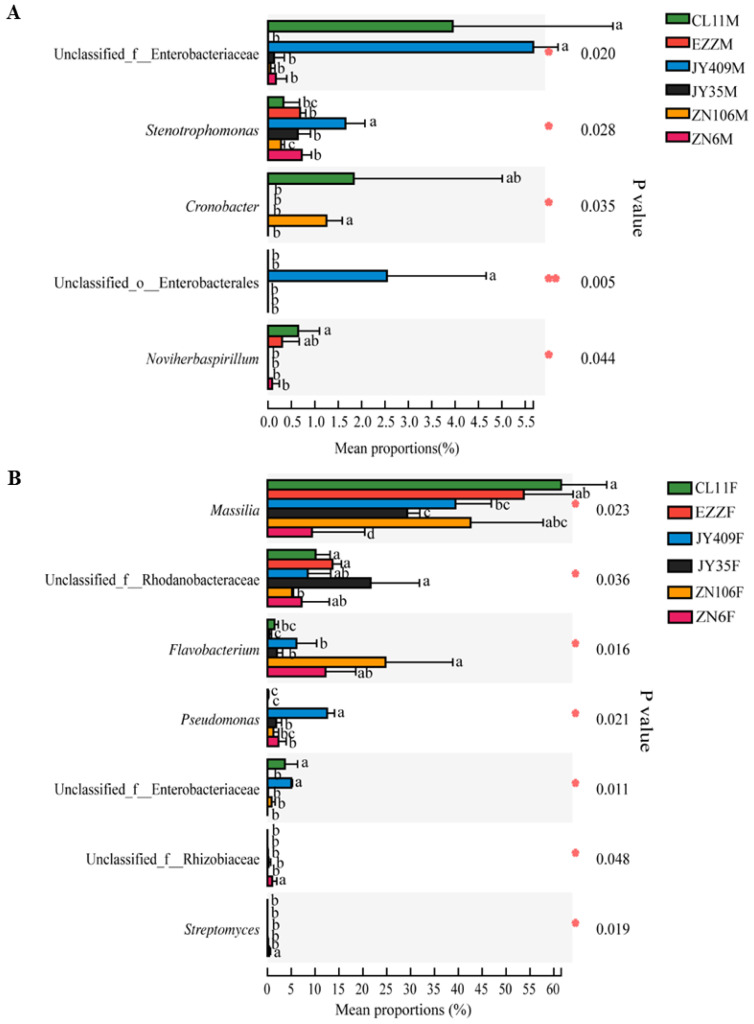
Differential bacterial genera across cucumber cultivars. The data were tested using the Kruskal-Wallis H test (*p* < 0.05) and validated with the false discovery rate (FDR) and Tukey-Kramer with a confidence level of 0.95. *, 0.01 < *p* ≤ 0.05; **, 0.001 < *p* ≤ 0.01. (**A**) Mock and (**B**) *Foc* inoculation.

Differential bacteria across cucumber cultivars were identified on phylum, genus, and ASV levels to reveal their co-association with cultivar resistance. As shown in Figure S4A, four phyla were found to be significantly different (*p* < 0.05) in the root microbiota of cucumber. The relative abundance of Enterobacteriaceae was higher in resistant cultivars CL11, JY409 and ZN106, while the abundance of unclassified_o_Enterobacterales in JY409, Sphingobacteriaceae in JY35, and Moraxellaceae in ZN106 and ZN6 were relatively higher. At the genus level, five genera, including unclassified_f__Enterobacteriaceae, *Stenotrophomonas*, *Cronobacter*, unclassified_o__Enterobacterales, and *Noviherbaspirillum* showed significant differences (*p* < 0.05) in abundance across the cucumber cultivars, and unclassified_f__Enterobacteriaceae in CL11 and JY409, *Stenotrophomonas* in JY409, *Cronobacter* in CL11 and ZN106, unclassified_o__Enterobacterales in JY409, and *Noviherbaspirillum* in CL11 and EZZ were predominant (Figure 4A). However, at the ASV level, only four differential ASVs corresponding to *Cronobacter*, *Pseudomonas*, *Noviherbaspirillum*, and *Massilia* were identified (Table S6). Although the abundances were less than 2.0% across the cultivars, ASV162 (*Cronobacter*) in cultivar ZN106, ASV103 (*Pseudomonas*) in JY409, ASV124 (*Noviherbaspirillum*) in CL11, and ASV74 (*Massilia*) in ZN6 showed significantly higher abundance than that in other cultivars. These results suggested that only *Cronobacter* ASV162 of Enterobacteriaceae was consistently differential at the ASV, genus, and phylum levels.

Upon *Foc* inoculation, more bacterial phyla (six) and genera (seven) showed significant differences (*p* < 0.05) across the cucumber cultivars (Figure S4B and Figure 4B). At the phylum level, the relative abundance of Enterobacteriaceae was still higher in the root of the resistant cultivars CL11, JY409, and ZN106, while Pseudomonadaceae and Streptomycetaceae were only predominant in JY409 and ZN6, respectively. Rhodanobacteraceae, Flavobacteriaceae, and Xanthomonadaceae were differential, but their abundances in susceptible ZN6 were as high as those in some other resistant or moderately resistant cultivars, suggesting that these phyla might not be important bacteria related to cultivar resistance. At the genus level, *Pseudomonas* and unclassified_f__Enterobacteriaceae showed similar differential patterns to their corresponding phyla, showing higher abundance in resistant JY409, CL11 and JY409 than in susceptible ZN6, while the predominant *Streptomyces* in susceptible ZN6 also showed a similar pattern to its corresponding phylum. Distinct from the genera described above, *Massilia* was differential between the resistant and susceptible cultivars, but its corresponding phylum was not. Another two differential bacteria between the resistant and susceptible cultivars were unclassified_f__Rhizobiaceae and *Streptomyces*, whose abundances were higher in susceptible ZN6. Further analyses at the ASV level showed that the two ASVs of *Massilia* (ASV1 and ASV4) were differential across cultivars (Table S6). ASV1 dominated in all the resistant cultivars with proportions of 23.12–52.55%, but it only accounted for 5.36% of the bacterial community of susceptible ZN6. ASV4 showed similar patterns to ASV1, but its abundance was much lower in the resistant and susceptible cultivars (2.75–6.41% vs. 0.55%). Some differential bacterial ASVs also showed co-association with specific cultivars, such as *Pseudomonas* (ASV48 and ASV103), dominant in JY409, unclassified_f__Rhodanobacteraceae (ASV33), dominant in EZZ, and *Flavobacterium* (ASV95), dominant in ZN106. Upon *Foc* inoculation, only Pseudomonadaceae/*Pseudomonas*/ASV103 and ASV48 were differential at the ASV, genus, and phylum levels, while unclassified_f__Enterobacteriaceae was differential at the genus and phylum levels, and *Massilia* was differential at the ASV and genus levels.

The results altogether suggested that each cucumber cultivar with different CFW resistance levels had specific root microbiota characterized by the differential assembly of bacteria. In response to resistance variation and pathogenic attack, *Massilia*, unclassified_f_Enterobacteriaceae, *Pseudomonas*, and *Cronobacter* were identified as important bacteria that might play important roles in cucumber resistance against *Foc*.

### 3.5. Antagonistic Ability of the Culturable Bacterial Isolates

A total of 61 culturable bacteria were obtained by using two isolation methods, and 18 identified isolates (29.51%) based on the 16S rRNA V3-V4 sequences were unclassified using the culture-free method according to their corresponding ASV sequences (Table 2 and Table S7), but they were consistent at the family level. Among the 37 bacterial isolates obtained from the cucumber roots using the bacterial band method (Table 2), 19 isolates were confined into three genera of Enterobacteriaceae, including *Enterobacter*, *Pantoea*, and *Cronobacter*. Two isolates of *Enterobacter* spp. (E307 and E317) and two isolates of *Pantoea* (P304 and E318), corresponding to unclassified Enterobacter ASV 343, were obtained from resistant cultivars EZZ, JY35, JY409, and CL11, and showed different antagonistic activities (19.83–41.84%) against *Foc*. Furthermore, eight isolates of *Cronobacter* spp., corresponding to ASV 162, were specifically isolated from moderately resistant cultivar ZN106 and showed higher inhibitory rates of 35.50–55.71%. Using the conventional plating method, only 24 bacterial isolates belonging to 10 genera were obtained from cultivars CL11, JY409, and ZN6, and their antagonistic activities against *Foc* ranged from 13.34% to 35.50%. Of them, four isolates (P25, P26, P28, and P29) of *Pantoea*, corresponding to ASV410, were identified as unclassified_o__Enterobacterales (Table S7), a differential genus with significantly high abundance in JY409 (Figure 4). Three isolates, D38, D43, and Pa30, from cultivars CL11, JY409, and ZN6, respectively, were assigned to *Roseomonas* based on the 16S rRNA V3-V4 sequences, which was inconsistent with the taxa (*Devosia*) identified with the culture-free method (a low homology of 87.4%).

### 3.6. Suppression of CFW by Differential Bacteria

**Figure 5:**
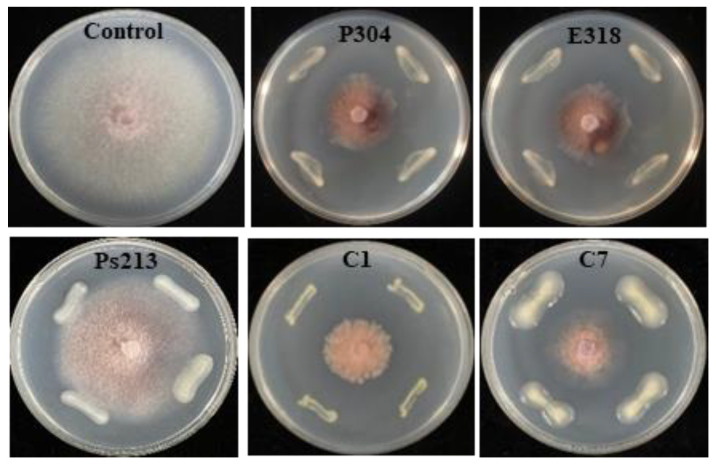
Antagonism of five differential bacteria on the hyphae growth of *Foc*.

Based on the construction of the phylogenetic trees of five bacteria, strains P304 and E318 were identified as *Pantoea dispersa*, evidenced by the homology of 100% shown in Figure S5. Strain Ps213 was classified as *Pseudomonas koreensis* (Figure S6), but strains C1 and C7 could only be designated as *Cronobacter* spp. (Figure S7) because they showed the highest homology to both *Cronobacter malonaticus* and *C*. *sakazakii*. The *in vitro* antagonistic tests showed that *P*. *dispersa* strains P304 and E318 and *Cronobacter* spp. strains C1 and C7 not only suppressed *Foc* growth but also altered *Foc* colony morphology, demonstrated by the brick-red change in colony color. In contrast, *P. koreensis* Ps213 did not show a suppressing ability against *Foc* growth (Figure 5). The further potting experiment showed that all the test bacteria alleviated the disease severity and the disease incidence of CFW except for *P. dispersa* strain E318, which only alleviated the disease index (*p* < 0.05; Table 3). The bacterial complexes BCX1 (*P*. *dispersa* E318 and *P. koreensis* Ps213) and BCX2 (*Cronobacter* C1 and C7) showed higher control efficacies (77.2% and 60.0%, respectively) than a single bacterium. The results indicated that some strains of *Pantoea*, *Pseudomonas*, and *Cronobacter* conferred protection against *Foc* infection in cucumber (*p* < 0.05) alone and in combination.

## 4. Discussion

Plant microbiota interacts with plant hosts through some functional traits to improve plant fitness [44]. The rhizospheric bacterial community was mainly shaped with the root exudates, which widely differed in the composition and amount exerted by the plant species or plant genotypes with variable stress resistance [19,45,46]. To reveal the relationship between cultivar resistance and root microbiota, we selected six northern and southern cucumber cultivars of different resistance against CFW. Of them, the resistance of cultivars JY409, JY35, ZN106, and ZN6 were consistent with previous studies [26,27,28,29], suggesting that the resistance evaluation method used in this study was effective and reliable. By using this method, cultivars CL11 and EZZ were also defined as resistant cultivars against CFW. These cultivars with distinct physiological characteristics were then grown in soil inoculated with *Foc* to clarify whether the root bacterial communities were shaped by cultivars or pathogens.

### 4.1. Specific Assembly of Root Microbiota Associated with Cultivars and Pathogens

The composition of the rhizospheric microbial community is shaped by plant genotypes as well as biotic and abiotic stresses. Besides rhizospheric bacteria, the root microbiota also closely interacts with plants, but their compositions and functions are distinctly different [47]. Previous studies on the rhizospheric bacterial communities of resistant and susceptible cucumber cultivars grown in the field suggested that the enrichment of specific rhizospheric bacteria is related to the genotype and the resistance of cultivars [21,23,48]. In this study, the root bacterial community profiles of six cucumber cultivars were found to be different and varied in response to pathogenic attack (Figure 1, Figure 2 and Figure 3, Figure S3, and Table S4). Upon *Foc* inoculation, the predominant Oxalobacteraceae became differential between the resistant cultivars and susceptible ZN6, and the abundance was increased in resistant CL11, EZZ, and JY409, whereas it decreased in moderately resistant JY35 and ZN106 and susceptible ZN6. Besides Oxalobacteraceae, Enterobacteriaceae was predominant in resistant CL11, JY409, and ZN106, and unclassified_o_Enterobacterales and Pseudomonadaceae were predominant in JY409. These results obviously differed from previous field studies which showed that actinomycetes were enriched in the rhizosphere of resistant cultivars Zhongnong13 and Jinyou 3 [21] and susceptible cultivar Jinyan 4 [48], and Comamonadaceae and Xanthomonadacea were enriched in the rhizosphere of susceptible cultivar B80 [23]. Comamonadaceae and Xanthomonadacea were also detected in the root microbiota in this study, but their abundances had no significant variations upon *Foc* attack (Figure 1A). Different cultivars, soil, and sample sources might account for the inconsistency of previous and present studies.

Differential bacteria at the genus level were mainly concerned (Figure 4). *Massilia* which was reported as abundant in cucumber seeds and roots [49,50] was also found to be predominant in this study. The abundances of *Massilia* and unclassified_f_Oxalobacteraceae followed the same changing pattern (increased in the resistant cultivars) to their corresponding phylum, Oxalobacteraceae, upon *Foc* attack. Similarly, *Pseudomonas* and unclassified_f__Enterobacteriaceae showed the same changing patterns as their respective phyla (Figure 1, Table S2). This suggested that the abundance changes of some specific bacteria are associated with cultivar resistance and might be involved in the plant’s defense against pathogens.

### 4.2. Co-Association of Specific Bacteria with the Resistant Cucumber

Previous studies suggested that resistant cultivars may act synergistically with root-associated microorganisms, especially endophytic bacteria [51], against pathogenic fungi or bacteria [52,53,54]. During the processes of root bacterial community assembly, selection pressures drive the endophytic plant community to adapt and tolerate stress. Such interactions between plants and microorganisms render specific endophytes beneficial to their hosts [55,56]. Li et al. [47] found that the increased abundance and diversity of endophytic bacteria and the higher proportions of antagonistic bacteria, including *Rhizobium*, *Streptomyces*, *Pseudomonas*, *Pantoea*, and *Bacillus*, may contribute to the systematic defense of resistant peach cultivars against *Agrobacterium tumefaciens*. In this study, Enterobacteriaceae: *Cronobacter*: ASV162 and Pseudomonadaceae: *Pseudomonas*: ASV103/ASV48 were differential in non-*Foc* inoculated cultivars, while Oxalobacteraceae: *Massilia*: ASV1/ASV4 became differential upon *Foc* attack (Table S6), suggesting their key roles in the interactions of cucumber, root bacteria, and *Foc*. The co-association of Enterobacteriaceae, Pseudomonadaceae, unclassified_f__Enterobacteriaceae, unclassified_o_Enterobacterales, and *Pseudomonas* with resistant cultivars agreed with previous studies (Figure 4 and Figure S4) [18,20]. *Flavobacterium* was evidenced as a key bacterium related to the resistance of tomato wilt-resistant variety Hawaii 7996 [19]. In this study, *Flavobacterium* and unclassified_f_Rhodanobacteraceae were also found to be associated with some resistant cultivars (Figure 4B), but they were only differential at the ASV level between specific resistant cultivars and susceptible ZN6 (Figure 4B, Table S6). Thus, these taxa were excluded as key bacteria with resistance to CFW.

A profound understanding of the underlying mechanisms of the specific associations of root bacteria with plant resistance is important to reveal plant-microbiome interactions. Recent studies indicated that the co-association of plants and the microbiome depends on the specific plant metabolites or root exudates. For example, the *Arabidopsis* root microbiota was specifically modulated by the root triterpene [24]; the enrichment of *Massilia* was found to be related to the root flavones of maize [57]. *Enterobacter* and *Bacillus* were also associated with bitter triterpenoid-cucurbitacins in melon root [58], and the abundance of Enterobacteriaceae increased after the application of L-asparagine and L-glutamic acid in the rhizosphere [59]. A pathogenic attack may trigger the plant defense pathway, which further shapes the root microbiota and enriches some functional bacteria. Proteobacteria, Actinobacteria, and Firmicutes were associated with the salicylic acid pathway in *Arabidopsis* [60]. The enrichment of Enterobacteriaceae in the *myc2*-depleted mutant of *Arabidopsis* [61] suggested that Enterobacteriaceae was related to the jasmonate-dependent defense. We thus infer that the resistant and susceptible cucumber cultivars vary in the pathogen-induced biosynthetic pathways that encode specific defense-related molecules, including the production of specific signals and root exudates, the up-regulation of specific genes, and the enrichment of specific corresponding root bacteria.

### 4.3. Enhanced Resistance Exerted by Differential Bacteria

The functions of rhizospheric microbes were tested using the approaches of microbes’ transmission, community transplantation, and synthetic community [19,47,62]. For example, Kwak et al. (2018) transplanted the enriched *Flavobacterium* in the resistant tomato variety Hawaii 7996 and improved the resistance of the susceptible variety moneymaker to *Ralstonia solanacearum*. The antagonistic activities of the cultivable root bacteria alone or in a complex were also tested using a conventional bioassay method. In this study, more antagonistic strains of *Enterobacter*, *Cronobacter*, *Pantoea*, and *Pseudomonas* were isolated from the resistant cultivars CL11, JY409, and ZN106 than susceptible ZN6 (Table 2) and fulfill important functions in suppressing *Foc* infection or reducing *Foc* pathogenicity (Table 3). Among those culturable bacteria, *Cronobacter* spp. C1 and C7 were identified as the differential bacteria at the phylum, genus, and ASV levels, while *Pseudomonas koreensis* Ps213 and *Pantoea dispersa* P304 and E318 were identified as differential bacteria at the genus level. In addition, the biocontrol capabilities of *P. koreensis* in suppressing *Phytophthora infestans* and *Pythium ultimum* through producing biosurfactants [63,64] and *P. dispersa* in suppressing the *Fusarium* wilt of pigeon pea [65] and the black rot of sweet potato were reported [66]. In this study, strains C1, C7, P304, and E318 not only strongly inhibited the hyphae growth of *Foc*, but also changed its colony morphology. It indicated that these antagonistic bacteria might function through secondary metabolites instead of direct colonization. *P. koreensis* Ps213 had relatively low antagonistic activity on the plate (Figure 5, Table S7), but strongly alleviated CFW alone and in a complex with *P. dispersa* E318 (complex BCX1). It is interesting that *P. dispersa* may produce salicylic acid, benzene acetic acid, and other organic acids to dissolve insoluble phosphorus and activate soil microbial communities [67]. It is well known that salicylic acid is a chemical that induces plant resistance against pathogenic attacks. Thus, the bacterial complex BCX1 might suppress CFW disease through systemic acquired resistance, and this needs further research.

The isolation of bacteria and the screening of antagonistic strains is important for functional confirmations and applications in the field. In this study, the bacterial band method was used to isolate antagonistic bacteria directly from cucumber root bacterial suspension, which was used to test bacterial colonization on a fungal hyphosphere [30,42]. This method was effective, with a high selecting efficiency of 51% (Table 2). However, some bacteria were difficult to isolate or were uncultivable. *Massilia* was most predominant and differential after *Foc* infection, but no *Massilia* strains were successfully isolated in this study. The reasons might be that *Massilia* in cucumber root is different from the species isolated from forest soil by Altankhuu et al. [68] or is in a non-cultivable state stressed by an extremely acidic environment [47,69]. The culturomics technique may be used to obtain the target microbes and facilitate functional studies [70].

## 5. Conclusions

In conclusion, different cucumber cultivars had similar root bacterial communities, but the interaction of cucumber with *Foc* obviously shifted the bacterial community. The bacterial community shifts were related to the cultivars and their resistance rather than the resistant level. Differential bacteria Enterobacteriaceae (including *Pantoea*, *Enterobacter,* and *Cronobacter*) and *Pseudomonas* associated with resistant CL11 and JY409, and moderately resistant ZN106 played important roles in suppressing CFW disease, and the bacterial complex *Pantoea dispersa + Peudomonas koreensis* and *Cronobacter* have great potential to be applied in the management of CFW disease.

## Figures and Tables

**Table 1:** Alpha diversity of the bacterial microbiota of cucumber roots at the ASV level ^a^.

Treatment	Cucumber Cultivar	Good’s Coverage	Chao	Shannon	Simpson
Mock	CL11	1.00 ± 0.00	43.00 ± 12.49	2.40 ± 0.18	0.18 ± 0.02
	EZZ	1.00 ± 0.00	44.33 ± 4.04	2.29 ± 0.31	0.21 ± 0.08
	JY409	1.00 ± 0.00	43.33 ± 4.62	2.87 ± 0.16	0.10 ± 0.02
	JY35	1.00 ± 0.00	48.67 ± 11.02	2.64 ± 0.46	0.17 ± 0.11
	ZN106	1.00 ± 0.00	42.00 ± 11.53	2.27 ± 0.16	0.04 ± 0.20
	ZN6	1.00 ± 0.00	53.33 ± 10.50	2.44 ± 0.10	0.20 ± 0.00
*Foc* inoculation	CL11	1.00 ± 0.00	41.33 ± 4.04	2.05 ± 0.23	0.30 ± 0.08
	EZZ	1.00 ± 0.00	44.33 ± 8.51	2.38 ± 0.37	0.21 ± 0.09
	JY409	1.00 ± 0.00	47.67 ± 16.17	2.68 ± 0.37	0.15 ± 0.05
	JY35	1.00 ± 0.00	50.33 ± 6.66	2.83 ± 0.16	0.12 ± 0.03
	ZN106	1.00 ± 0.00	41.67 ± 5.13	2.31 ± 0.04	0.21 ± 0.01
	ZN6	1.00 ± 0.00	54.67 ± 12.90	2.65 ± 0.43	0.15 ± 0.07

**^a^** The data have no significant difference based on Duncan’s multiple-range test (*p* > 0.05).

**Table 2:** Culturable bacteria isolated from the cucumber roots using the bacterial band method.

Cucumber Cultivar	Isolate	Inhibition Rate (%)	Taxa Based on the 16S rRNA Gene Sequence	Taxa and ASVs Based on the Illumina High-Throughput Analysis		Homology (%)
				Genus	ASV	
CL11	E11	20.40 ± 1.37	*Enterobacter*	Unclassified_f__Enterobacteriaceae	ASV341	100.0
	Pa301	28.57 ± 3.80	*Paenibacillus*	*Paenibacillus*	ASV62	93.7
	P300	29.89 ± 2.88	*Pantoea*	Unclassified_f__Enterobacteriaceae	ASV478	96.8
	S305	8.41 ± 2.44	*Sphingomonas*	*Sphingomonas*	ASV463	99.7
	Ag302	18.82 ± 0.77	*Rhizobium*	Unclassified_c__Gammaproteobacteria	ASV53	94.0
	Br303	27.11 ± 1.37	*Brucella*	Unclassified_f__Rhizobiaceae	ASV298	95.8
	P304	41.84 ± 0.31	*Pantoea*	*Enterobacter*	ASV343	98.4
EZZ	B312	16.39 ± 4.04	*Bordetella*	Unclassified_f__Alcaligenaceae	ASV156	99.7
	A308	12.88 ± 1.96	*Agrobacterium*	*Allorhizobium-Neorhizobium-Pararhizobium-Rhizobium*	ASV53	94.0
	E307	19.83 ± 2.93	*Enterobacter*	*Enterobacter*	ASV343	100.0
	B309	18.75 ± 2.58	*Brucella/Ochrobactrum*	*Ochrobactrum*	ASV298	95.8
	B311	12.16 ± 4.17	*Bordetella*	Unclassified_f__Alcaligenaceae	ASV5	100.0
	B310	11.15 ± 1.35	*Bordetella*	Unclassified_f__Alcaligenaceae410	ASV5	100.0
JY409	E12	28.40 ± 0.89	*Enterobacter*	Unclassified_o__Enterobacterales	ASV410	99.7
	E318	41.14 ± 1.92	*Pantoea*	*Enterobacter*	ASV343	98.4
	E314	21.68 ± 6.29	*Enterobacter*	Unclassified_f__Enterobacteriaceae	ASV55	94.0
	E315	21.22 ± 5.39	*Enterobacter*	Unclassified_f__Enterobacteriaceae	ASV55	95.0
	E316	17.20 ± 0.96	*Enterobacter*	Unclassified_f__Enterobacteriaceae	ASV55	95.0
JY35	B13	21.30 ± 1.35	*Bordetella*	Unclassified_f__Alcaligenaceae	ASV5	100.0
	E317	31.85 ± 2.71	*Enterobacter*	*Enterobacter*	ASV343	100.0
	S313	22.75 ± 2.58	*Stenotrophomonas*	*Stenotrophomonas*	ASV46	99.7
	Br319	27.61 ± 0.07	*Brucella*/*Ochrobactrum*	*Ochrobactrum*	ASV298	95.8
ZN106	C1-C5, C7-C9	35.50 ± 5.00 ~ 55.71 ± 2.84	*Cronobacter*	*Cronobacter*	ASV162	100.0
ZN6	Ar10	15.20 ± 3.13	*Arachidicoccus*	*Arachidicoccus*	ASV439	99.5
	E14	37.20 ± 2.65	*Enterobacter*	Unclassified_f__Enterobacteriaceae	ASV55	99.7
	Ps20 -Ps24	17.10 ± 1.10	*Pseudomonas*	*Pseudomonas*	ASV263	99.7

**Table 3:** Control efficacies of the root bacterial isolates and their complexes against CFW in pots.

Bacteria ^a^	Species	Corresponding ASV	Disease Incidence (%) ^b^	Disease Index	Control Efficacy (%)
P304	*Pantoea dispersa*	ASV343	43.3 ± 8.17 ab	19.6 ± 5.20 a	46.3
E318	*P. dispersa*	ASV343	50.0 ± 0.00 ac	20.8 ± 12.50 a	42.9
Ps213	*Pseudomonas koreensis*	ASV466	41.7 ± 16.67 ab	15.6 ± 3.99 a	57.1
BCX1	*P. dispersa* + *P. koreensis*	ASV343+ASV466	29.2 ± 4.81 b	8.3 ± 2.95 b	77.2
BCX2	*Cronobacter*	ASV162	29.2 ± 15.96 b	14.6 ± 2.41 ab	60.0
Control	-	-	66.7 ± 13.61 c	36.5 ± 6.25 c	-

^a^ BCX1 and BCX2 were the bacterial complexes of strains E318 and Ps213 and strains C1 and C7 respectively, mixed at a volume ratio of 1:1. ^b^ Different letters following the data showed significant differences (*p* < 0.05) based on Duncan’s multiple-range test.

## Data Availability

All data were published in this article.

## Supplementary Materials

The following supporting information can be downloaded at: https://www.mdpi.com/article/10.3390/biology12020143/s1. The following are available online, Figure S1: Demonstration of the isolation strategy of antagonistic bacteria. Figure S2: Demonstration of *Foc* inoculation site to the cucumber seedlings grown in bacteria inoculated soil. Figure S3: Taxonomic profiling of the bacterial communities in cucumber root across cultivars and treatments at phylum level. Phyla with a proportion of less than 1.0% are combined into the group “Others”. CL11, EZZ and JY409 are resistant cultivars, JY35 and ZN106 are moderately resistant cultivars, and ZN6 is a susceptible cultivar. M and F indicates mock and *Foc* inoculation, respectively. Figure S4: Differential bacterial phyla among cucumber cultivars with mock (A) and *Foc* inoculation (B). Data were tested by Kruskal-Wallis H test (*p* < 0.05) and validated by False Discovery Rate (FDR) and Tukey-Kramer with a confidence level of 0.95. *, 0.01 < *p* ≤ 0.05; **, 0.001 < *p* ≤ 0.01. Different letters beside the error bars of each phylum indicate significant difference among cultivars (*p* < 0.05). Figure S5: Phylogenetic trees of strains P304 and E318 based on their 16S rRNA gene sequences determined by the neighbor-joining method with the program package MEGA 11.0. Bootstrap confidence values were obtained using 2000 resamplings. Bar, 0.01 substitutions per site. Figure S6: Phylogenetic tree of strain Ps213 based on its 16S rRNA gene sequence determined by the neighbor-joining method with the program package MEGA 11.0. Bootstrap confidence values were obtained using 2000 resamplings. Bar, 0.02 substitutions per site. Figure S7: Phylogenetic trees of strains C1 and C7 based on their 16S rRNA gene sequences determined by the neighbor-joining method with the program package MEGA 11.0. Bootstrap confidence values were obtained using 2000 resamplings. Bar, 0.01 substitutions per site. Table S1: Resistance of cucumber cultivars to CFW. Table S2: Relative abundance of the predominant genera (>5.00%) in cucumber root. Table S3: Comparison of the relative abundance of *Massilia* and unclassified Oxalobacteraceae against the whole family Oxalobacteraceae in the cucumber root microbiota. Table S4: Permutational multivariate analysis of variance (PERMANOVA) of the root bacterial microbiota of cucumber roots with (Group F) or without *Foc* (Group M) inoculation based on Bray-Curtis dissimilarity and 999 permutations. Table S5: Analysis of similarity (ANOSIM) of the cucumber cultivars with mock (M) or *Foc* inoculation (F). Table S6: Differential bacterial ASV across cucumber cultivars inoculated with mock or *Foc* inoculation. Table S7: Isolation and identification of the culturable bacteria isolated from cucumber roots by using the conventional plating method.
