# Supplementary material for: The Co-Association of Enterobacteriaceae and Pseudomonas with Specific Resistant Cucumber against Fusarium Wilt Disease
Source: Biology (Basel). 2023 Jan 17;12(2):143. doi: 10.3390/biology12020143 (PMC9952826; doi:10.3390/biology12020143)
Supplement: Supplementary file 1 [file biology-12-00143-s001.zip › biology-2158008-Supplementary files-2023-1-29.pdf]

biology

MDPI

*Supplementary Materials*

# The Co-Association of Enterobacteriacea and *Pseudomonas* with Specific Resistant Cucumber against *Fusarium* Wilt Disease

**Yu-Lu Zhang, Xiao-Jing Guo, Xin Huang, Rong-Jun Guo, Xiao-Hong Lu and Shi-Dong Li, Hao Zhang**

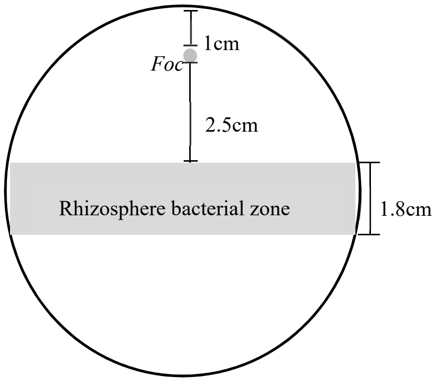


**Figure S1.** Demonstration of the isolation strategy of antagonistic bacteria.

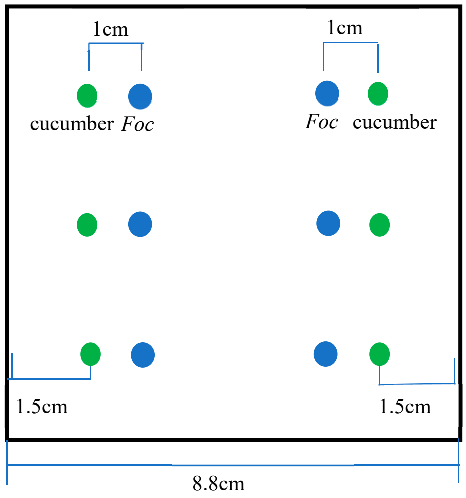


**Figure S2.** Demonstration of *Foc* inoculation site to the cucumber seedlings grown in bacteria inoculated soil.

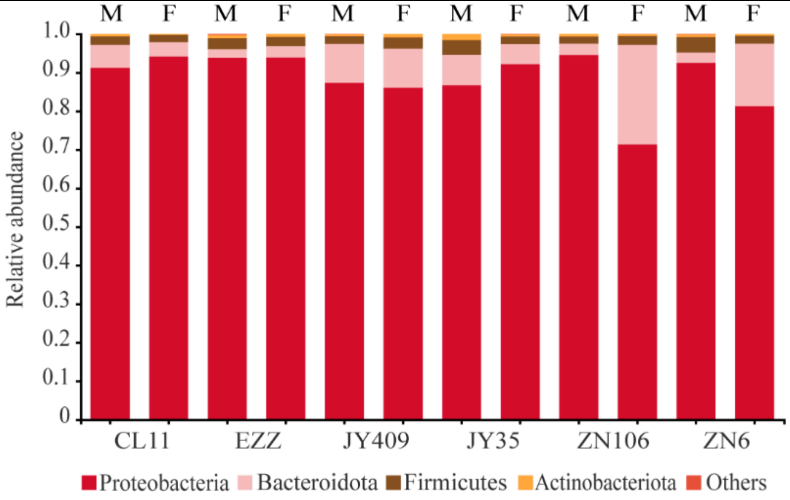


**Figure S3.** Taxonomic profiling of the bacterial communities in cucumber root across cultivars and treatments at phylum level. Phyla with a proportion of less than 1.0% are combined into the group "Others". CL11, EZZ and JY409 are resistant cultivars, JY35 and ZN106 are moderately resistant cultivars, and ZN6 is a susceptible cultivar. M and F indicate mock and *Foc* inoculation, respectively.

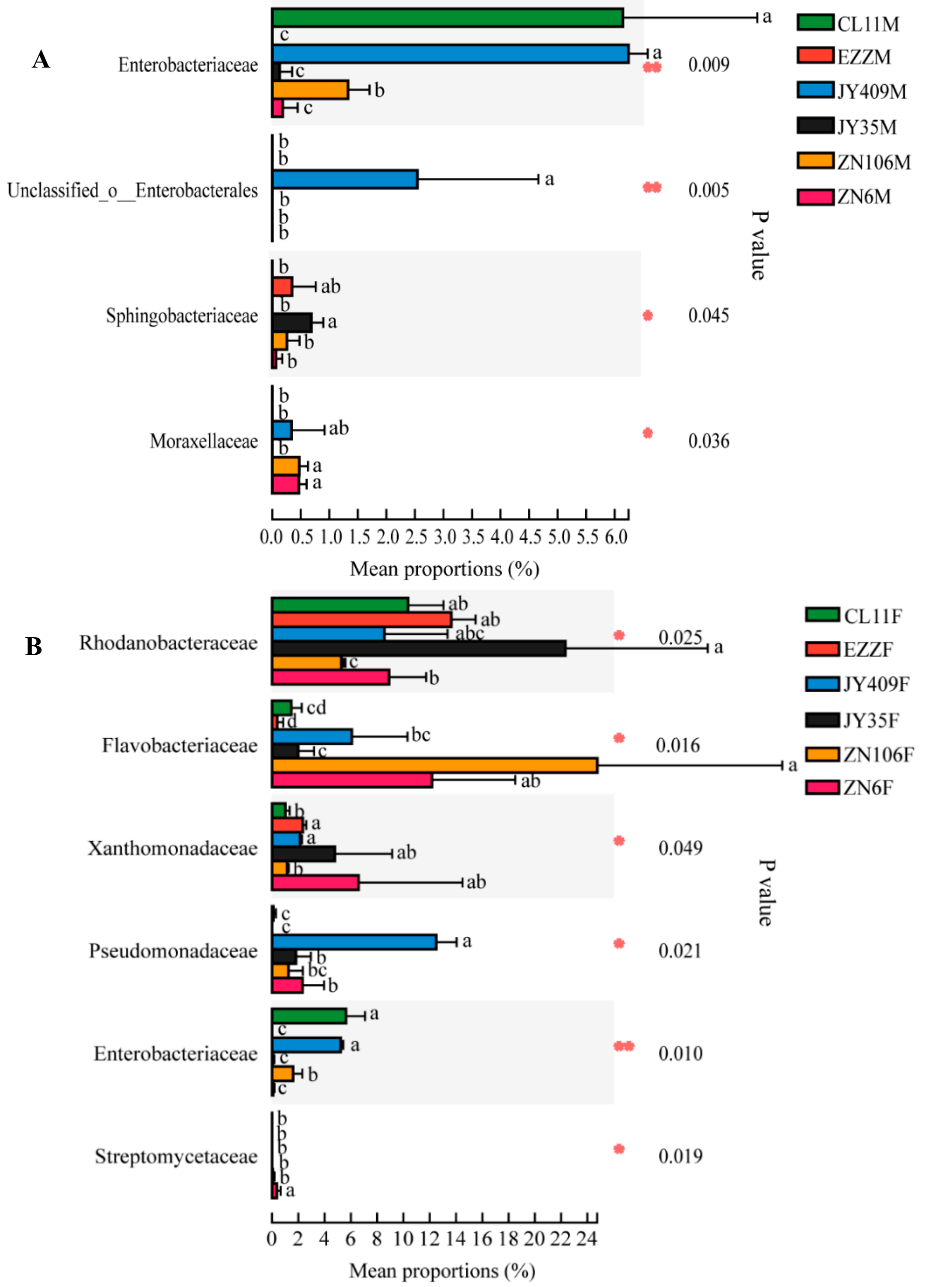


**Figure S4.** Differential bacterial phyla among cucumber cultivars with mock (A) and *Foc* inoculation (B). Data were tested by Kruskal-Wallis H test ($p < 0.05$) and validated by False Discovery Rate (FDR) and Tukey-Kramer with a confidence level of 0.95. *, $0.01 < p \leqslant 0.05$; **, $0.001 < p \leqslant 0.01$. Different letters beside the error bars of each phylum indicate significant difference among cultivars ($p < 0.05$).

biology MDPI

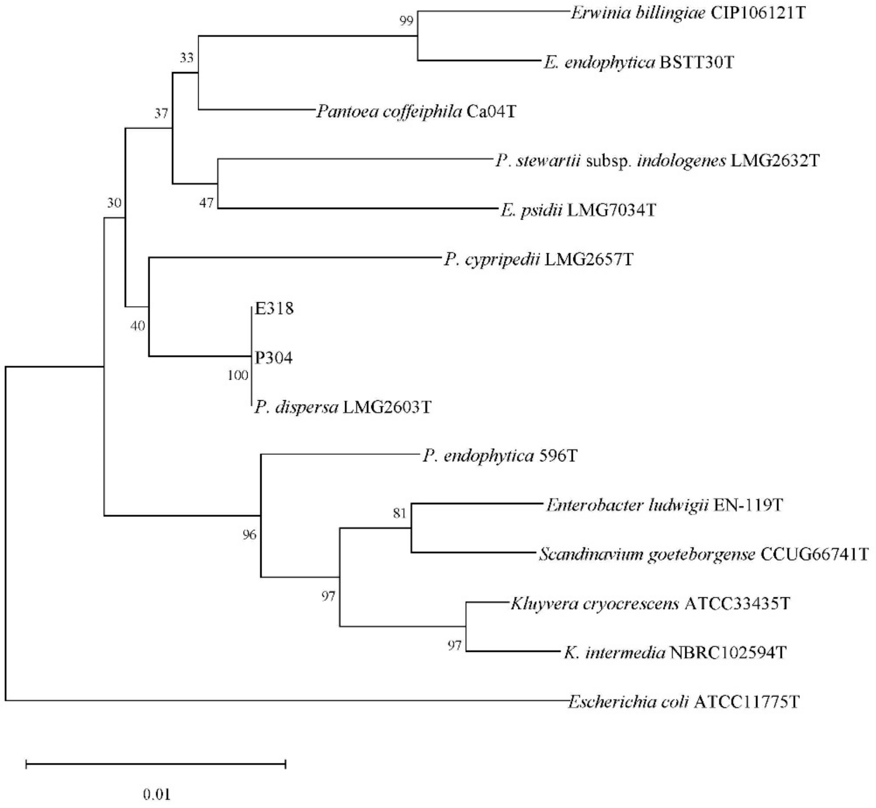


**Figure S5.** Phylogenetic trees of strain P304 and E318 based on their 16S rRNA gene sequences determined by the neighbor-joining method with the program package MEGA 11.0. Bootstrap confidence values were obtained using 2000 resamplings. Bar, 0.01 substitutions per site.

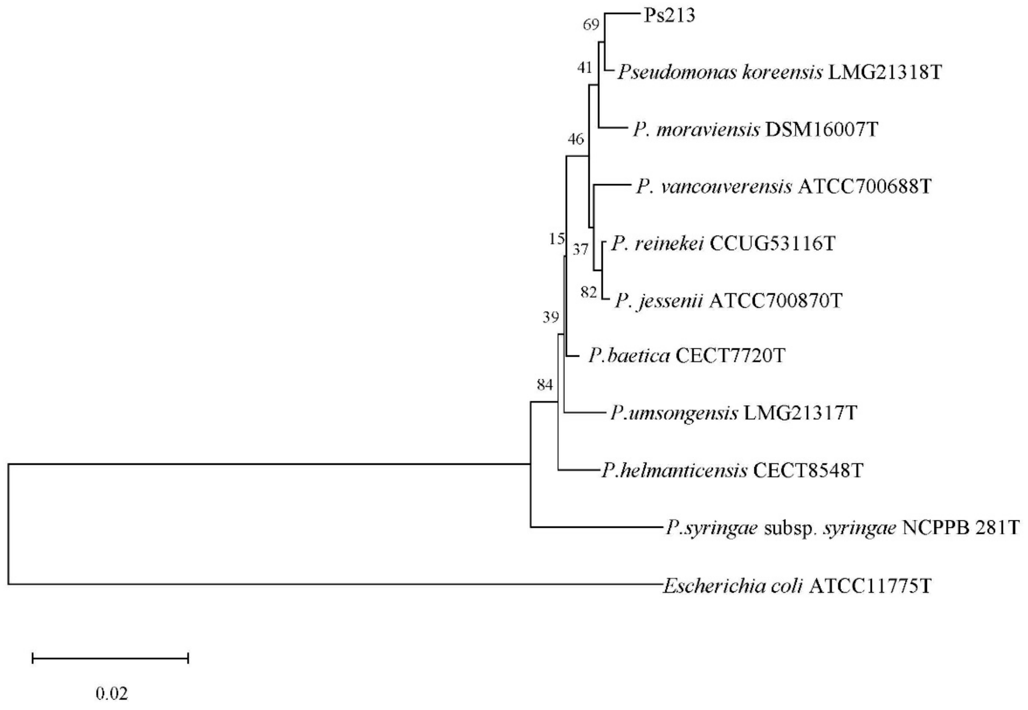


**Figure S6.** Phylogenetic tree of strain Ps213 based on its 16S rRNA gene sequence determined by the neighbor-joining method with the program package MEGA 11.0. Bootstrap confidence values were obtained using 2000 resamplings. Bar, 0.02 substitutions per site.

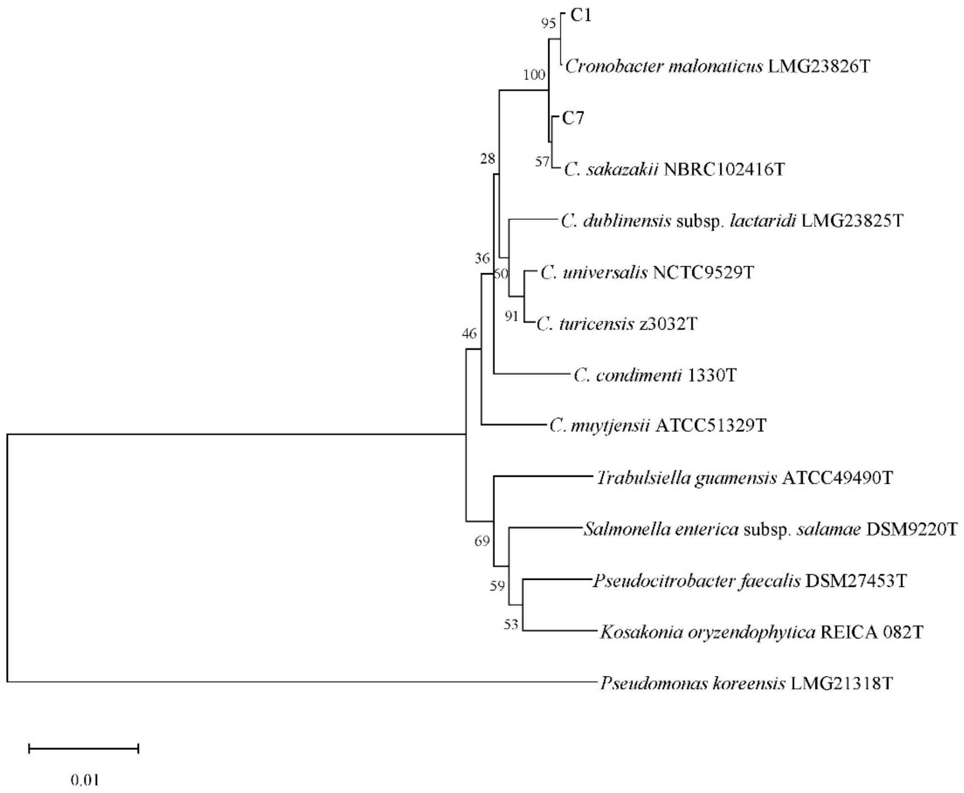


**Figure S7.** Phylogenetic trees of strain C1 and C7 based on their 16S rRNA gene sequences determined by the neighbor-joining method with the program package MEGA 11.0. Bootstrap confidence values were obtained using 2000 resamplings. Bar, 0.01 substitutions per site.

**Table S1.** Resistance of cucumber cultivars to CFW[a].

| Cucumber cultivar | Disease incidence (%) | | Disease index | | Resistance |
|---|---|---|---|---|---|
| CL11 | 0.00 ± 0.00 | a | 0.00 ± 0.00 | a | Resistant |
| EZZ | 0.00 ± 0.00 | a | 0.00 ± 0.00 | a | Resistant |
| JY409 | 16.67 ± 0.00 | abc | 8.32 ± 4.17 | ab | Resistant |
| JY35 | 38.89 ± 9.62 | c | 23.61 ± 6.36 | b | Moderately resistant |
| ZN106 | 33.34 ± 28.87 | bc | 15.27 ± 15.78 | ab | Moderately resistant |
| ZN6 | 88.89 ± 19.24 | d | 51.39 ± 17.35 | c | Susceptible |

[a] Different letters of the same column showed significant difference at $p < 0.05$ based on the Duncan’s new multiple-range test

**Table S2.** Relative abundance of the predominant genera (>5.00%) in cucumber root.

| **Cultivar** | Treatment | The relative abundance (%) | | | | | | | | | |
|---|---|---|---|---|---|---|---|---|---|---|---|
| | | *Massilia* | Unclassified Oxalobacteraceae | Unclassified Rhodanobacteraceae | *Pseudomonas* | *Flavobacterium* | Unclassified Alcaligenaceae | *Shinella* | *Brevendimonas* | *Stenotrophomonas* | Unclassified Enterobacteriaceae |
| CL11 | Mock | 41.65 | 0.00 | 26.02 | 0.12 | 4.31 | 3.83 | 3.54 | 0.81 | 0.33 | 3.95 |
| EZZ | | 47.83 | 0.75 | 21.9 | 0.28 | 1.06 | 7.31 | 4.1 | 1.26 | 0.69 | 0.00 |
| JY409 | | 28.08 | 0.13 | 13.08 | 13.86 | 6.56 | 5.89 | 3.83 | 1.56 | 1.66 | 5.67 |
| JY35 | | 39.71 | 0.92 | 15.40 | 0.15 | 4.28 | 9.33 | 7.56 | 1.24 | 0.64 | 0.13 |
| ZN106 | | 56.49 | 0.87 | 18.80 | 0.00 | 1.31 | 5.4 | 3.68 | 1.45 | 0.28 | 0.05 |
| ZN6 | | 49.77 | 1.12 | 20.03 | 0.53 | 0.66 | 5.06 | 4.15 | 1.7 | 0.72 | 0.16 |
| CL11 | *Foc* inoculation | 61.55 | 0.12 | 10.09 | 0.11 | 1.47 | 4.75 | 4.89 | 0.57 | 1.02 | 3.67 |
| EZZ | | 53.73 | 0.05 | 13.64 | 0.00 | 0.4 | 7.5 | 6.61 | 1.94 | 1.04 | 0.00 |
| JY409 | | 39.42 | 0.16 | 8.47 | 12.51 | 6.07 | 5.68 | 3.46 | 2.3 | 1.3 | 5.02 |
| JY35 | | 29.29 | 0.30 | 21.63 | 1.82 | 1.99 | 8.73 | 6.19 | 3.25 | 4.45 | 0.05 |
| ZN106 | | 42.57 | 0.36 | 5.18 | 1.25 | 24.75 | 5.83 | 4.87 | 2.58 | 0.94 | 0.88 |
| ZN6 | | 9.36 | 25.22 | 7.16 | 2.32 | 12.17 | 7.85 | 7.04 | 5.89 | 5.03 | 0.00 |

**Table S3.** Comparison of the relative abundance of *Massilia* and unclassified Oxalobacteraceae against the whole family Oxalobacteraceae in the cucumber root microbiota.

| **Cultivar** | Treatment | The relative abundance (%) | | |
|---|---|---|---|---|
| | | Oxalobacteraceae | *Massilia* + unclassified Oxalobacteraceae | Proportions (%) |
| CL11 | Mock | 42.29 | 41.65 | 98.49 |
| EZZ | | 48.88 | 48.58 | 99.39 |
| JY409 | | 28.20 | 28.21 | 100.04 |
| JY35 | | 40.63 | 40.63 | 100.00 |
| ZN106 | | 57.36 | 57.36 | 100.00 |
| ZN6 | | 50.98 | 50.89 | 99.82 |
| CL11 | *Foc* inoculation | 61.80 | 61.67 | 99.79 |
| EZZ | | 53.82 | 53.78 | 99.93 |
| JY409 | | 39.71 | 39.58 | 99.67 |
| JY35 | | 29.77 | 29.59 | 99.40 |
| ZN106 | | 42.92 | 42.93 | 100.02 |
| ZN6 | | 34.61 | 34.58 | 99.91 |

**Table S4.** Permutational multivariate analysis of variance (PERMANOVA) of the root bacterial microbiota of cucumber roots with (Group F) or without *Foc* (Group M) inoculation based on Bray-Curtis dissimilarity and 999 permutations.

| Source | Df | Sums of Sq | Mean Sq | F Model | $R^2$ | Pr (> F) |
|---|---|---|---|---|---|---|
| Group M | | | | | | |
| Cultivar | 5 | 0.86 | 0.17 | 1.25 | 0.34 | 0.156 |
| Residuals | 12 | 1.65 | 0.14 | | 0.66 | |
| Total | 17 | 2.51 | | | 1 | |
| Group F | | | | | | |
| Cultivar | 5 | 1.72 | 0.34 | 2.64 | 0.52 | 0.001 |
| Residuals | 12 | 1.56 | 0.13 | | 0.48 | |
| Total | 17 | 3.28 | | | | |
| Groups M & F | | | | | | |
| Cultivar | 5 | 1.57 | 0.31 | 2.16 | 0.26 | 0.001 |
| Inoculation | 1 | 0.25 | 0.25 | 1.75 | 0.04 | 0.040 |
| Residuals | 29 | 4.22 | 0.15 | | 0.70 | |
| Total | 35 | 6.04 | | | 1.00 | |

**Table S5.** Analysis of similarity (ANOSIM) of the cucumber cultivars with mock (M) or *Foc* inoculation (F).

| Paired groups | R value | *p* value |
|---|---|---|
| CL11M vs. CL11F | 0.44 | 0.10 |
| EZZM vs. EZZF | 0.04 | 0.51 |
| JY409M vs. JY409F | –0.04 | 0.62 |
| JY35M vs. JY35F | 0.11 | 0.49 |
| ZN106M vs. ZN106F | 0.52 | 0.10 |
| ZN6M vs. ZN6F | 0.41 | 0.19 |

**Table S6.** Differential bacterial ASVs across cucumber cultivars with mock or *Foc* inoculation [a].

| Treatment | Differential ASV | Genus | Proportions (%) | | | | | | |
|---|---|---|---|---|---|---|---|---|---|
| | | | CL11 | EZZ | JY409 | JY35 | ZN106 | ZN6 | *p* value |
| Mock inoculation | ASV162 | *Cronobacter* | 1.75 ± 3.03 a | 0 ± 0 a | 0 ± 0 a | 0 ± 0 a | 1.25 ± 0.34 b | 0 ± 0 a | 0.03 |
| | ASV103 | *Pseudomonas* | 0 ± 0 a | 0 ± 0 a | 1.87 ± 0.36 b | 0 ± 0 a | 0 ± 0 a | 0 ± 0 a | 0.00 |
| | ASV124 | *Noviherbaspirillum* | 0.65 ± 0.46 a | 0.24 ± 0.41 ab | 0 ± 0 b | 0 ± 0 b | 0 ± 0 b | 0.09 ± 0.15 b | 0.05 |
| | ASV74 | *Massilia* | 0.02 ± 0.03 a | 0 ± 0 a | 0 ± 0 a | 0 ± 0 a | 0 ± 0 a | 0.15 ± 0.05 b | 0.01 |
| *Foc* inoculation | ASV1 | *Massilia* | 52.55 ± 7.44 a | 41.76 ± 11.21 ab | 32.96 ± 8.94 bc | 23.12 ± 3.15 c | 34.61 ± 12.93 bc | 5.36 ± 9.29 d | 0.03 |
| | ASV95 | *Flavobacterium* | 1.25 ± 0.94 a | 0.074 ± 0.13 a | 5.79 ± 4.19 a | 0.66 ± 1.14 a | 23.79 ± 13.80 b | 0.10 ± 0.17 a | 0.03 |
| | ASV4 | *Massilia* | 6.24 ± 2.82 a | 6.41 ± 2.03 a | 3.84 ± 0.31 ab | 2.75 ± 0.36 bc | 4.55 ± 0.94 ab | 0.55 ± 0.95 c | 0.02 |
| | ASV48 | *Pseudomonas* | 0.11 ± 0.20 a | 0 ± 0 a | 10.98 ± 1.02 b | 1.44 ± 1.40 ac | 1.11 ± 0.96 ac | 1.97 ± 1.03 c | 0.03 |
| | ASV103 | *Pseudomonas* | 0 ± 0 a | 0 ± 0 a | 1.53 ± 0.56 b | 0 ± 0 a | 0.14 ± 0.24 a | 0.16 ± 0.27 a | 0.03 |
| | ASV33 | Unclassified_f_ Rhodanobacteraceae | 0.02 ± 0.03 a | 0.11 ± 0.04 b | 0 ± 0 a | 0 ± 0 a | 0 ± 0 a | 0 ± 0 a | 0.01 |

[a] Data were analyzed by the Kruskal-Wallis H test and verified by FDR and Tukey-Kramer with the confidence at 0.95; different letters of the same row showed significant difference at $p < 0.05$.

**Table S7.** Isolation and identification of the culturable bacteria isolated from cucumber roots by using the conventional plating method.

| Cucumber cultivar | Isolates | Inhibitory rate (%) | Taxa based on 16S rRNA gene sequences | Taxa and ASVs based on Illumina high-throughput analysis | | |
|---|---|---|---|---|---|---|
| | | | | Genus | ASV | Homology (%) |
| CL11 | P25, P26, P28, P29 | 33.17 ± 2.93 ~ 37.77 ± 2.60 | *Pantoea* | Unclassified_o__Enterobacterales | ASV410 | 93.1–97.6 |
| | D34, D37, D39 | 17.78 ± 2.13 ~ 19.10 ± 1.60 | *Delftia* | *Delftia* | ASV104 | 100.0 |
| | S36 | 22.36 ± 2.36 | *Sphingomonas* | *Sphingomonas* | ASV329 | 98.7 |
| | D38 | 23.50 ± 5.32 | *Roseomonas* | *Devosia* | ASV497 | 87.4 |
| | S40 | 25.91 ± 3.45 | *Sphingomonas* | *Sphingomonas* | ASV19 | 100.0 |
| | S41 | 29.82 ± 3.29 | *Hephaestia* | *Hephaestia* | ASV548 | 99.4 |
| | S42 | 22.90 ± 2.78 | *Sphingomonas* | *Sphingomonas* | ASV375 | 97.4 |
| JY409 | D43 | 28.82 ± 3.01 | *Roseomonas* | *Devosia* | ASV497 | 87.4 |
| | Sh44 | 13.34 ± 1.13 | *Shinella* | *Shinella* | ASV40 | 100.0 |
| | Bo45 | 18.12 ± 2.65 | *Bosea* | *Bosea* | ASV290 | 100.0 |
| | Ps213 | 21.63 ± 4.41 | *Pseudomonas* | *Pseudomonas* | ASV466 | 99.7 |
| ZN6 | Ps15-Ps19 | 22.37 ± 2.61 ~ 25.20 ± 3.71 | *Pseudomonas* | *Pseudomonas* | ASV466 | 99.7 |
| | Pa30 | 17.33 ± 5.01 | *Roseomonas* | *Devosia* | ASV497 | 87.4 |
| | H31 | 24.21 ± 3.28 | *Sphingomonas* | Unclassified_f__Sphingomonadaceae | ASV105 | 100.0 |
| | D32 | 20.34 ± 1.22 | *Delftia* | *Delftia* | ASV104 | 100.0 |
